# COVID-19 vaccine hesitancy in Turkey: A systematic review and meta-analysis

**DOI:** 10.1017/S0950268823001875

**Published:** 2023-11-24

**Authors:** Bugra Taygun Gulle, Meryem Merve Oren, Tuba Dal

**Affiliations:** 1 Department of Public Health, Division of Epidemiology, Faculty of Medicine, Dokuz Eylül University, Izmir, Turkey; 2 Department of Public Health, Faculty of Medicine, Istanbul University, Istanbul, Turkey; 3 Department of Medical Microbiology, Faculty of Medicine, Ankara Yıldırım Beyazıt University, Ankara, Turkey

**Keywords:** COVID-19, Meta-analysis, prevalence, vaccine hesitancy, Turkey

## Abstract

This systematic review and meta-analysis aims to estimate the prevalence of coronavirus disease 2019 (COVID-19) vaccine hesitancy in Turkey, which can aid future health policies and strategies. A comprehensive search was conducted on various databases using keywords related to COVID-19 vaccine hesitancy in Turkey. Quality assessment was performed using Joanna Briggs Institute (JBI) checklist for prevalence studies. Data extraction was conducted. The random effect model (DerSimonian and Laird method) was used in pooled prevalence data analysis (95% confidence interval [CI]). A total of 1,072 articles were identified. After removing duplicates and excluding articles, 61 articles remained for bias assessment. Among these, 19 articles with low risk of bias were included in the review and meta-analysis. Total population included in the analysis was 15,164, vaccine hesitancy was 30.5% (95% Cl: 24.3–36.8%). Prevalence of the vaccine hesitancy was found to be 39.8% (95% Cl: 31.4–48.2%) in studies conducted before the initiation of vaccination, while in studies conducted after the commencement of vaccination, hesitancy was 20.4% (95% Cl: 12.9–28%). We suggest conducting high-quality studies in different populations to understand the level of vaccine hesitancy, as many of the previous studies have mainly focused on healthcare workers and students, and rest were community-based studies, which have generally shown high bias. Also, we suggest that early vaccination can reduce vaccine hesitancy.

## Introduction

Coronavirus disease 2019 (COVID-19) is an infectious disease caused by severe acute respiratory syndrome coronavirus 2 (SARS-CoV-2) [1]. As of April 2023, more than 750 million cases of COVID-19 have been diagnosed worldwide, with nearly 7 million deaths reported due to the virus [2]. Turkey has also been significantly affected, with more than 17 million confirmed cases and over 100,000 deaths due to COVID-19 [3]. Vaccination has emerged as the most promising prospect of controlling the pandemic since the development of effective vaccines [4]. The World Health Organization (WHO) recommends vaccinating at least 70% of the total populations in countries, including 100% of healthcare workers and vulnerable groups [5]. According to the Ministry of Health, 58 million individuals in Turkey have received at least one dose of COVID-19 vaccine, while 53 million have received at least two doses. Information on the vaccination status of vulnerable groups and healthcare workers is not currently available [6].

Despite vaccination being a major achievement in public health, vaccine hesitancy remains a global concern [7]. Just as in developed countries, vaccine hesitancy has been increasing in developing countries such as Turkey in recent years [8]. COVID-19 vaccine hesitancy appears to be no exception [9]. COVID-19 vaccine hesitancy and acceptance rates vary across countries and continents worldwide, with factors such as vaccine efficacy, safety, and trust in the government influencing individuals’ decision to receive the vaccine [10].

Systematic reviews and meta-analyses have been conducted in different countries regarding COVID-19 vaccine hesitancy [11–16]. Studies in Turkey have shown that COVID-19 vaccine hesitancy ranges from 2% to 98% [17, 18]. However, there is no systematic review and meta-analysis on this subject in Turkey. Therefore, in this systematic review and meta-analysis, we aimed to estimate the accurate proportion of COVID-19 vaccine hesitancy in Turkey. This study will provide valuable insights to guide evidence-based public health policies for policy makers, healthcare professionals, and stakeholders.

## Methods

### Protocol and registration

The protocol of this systematic review and meta-analysis was registered with the International Prospective Register of Systematic Reviews (PROSPERO) on 21 April 2023 (CRD42023418992). This registration ensures transparency and reduces the risk of bias in the study. Furthermore, adherence to Preferred Reporting Items for Systematic Reviews and Meta-Analysis (PRISMA) guidelines guarantees the comprehensive and accurate reporting of our research methodology [19].

### Search strategy

A comprehensive literature search was conducted using the following databases: PubMed, Web of Science, ProQuest, EBSCO, and TUBITAK-ULAKBIM. The following keywords were used in the research: “COVID-19”, “SARS-COV-2”, “vaccine*”, “hesitancy”, “acceptance”, “willingness”, “Turkey”. “And” and “OR” Boolean logic operators were employed to integrate the keywords. The electronic databases were searched on 22 April 2023.

### Selection criteria

Studies that met the following criteria were included in our study:

- Articles addressing COVID-19 vaccine hesitancy in adults and providing quantitative outcomes.
- Articles with participants aged over 18.
- Studies conducted in Turkey.
- Articles written in English or Turkish.
- Peer reviewed, original and published articles.
- Articles with full-text available.
- Studies with low bias risk.

For the purpose of our study, “vaccine hesitancy” is defined as reluctance or refusal to get vaccinated [20].

### Selection process

Following the article search process, duplicate articles were removed. After this step, two authors (B.T.G. and M.M.Ö.) independently and blindly screened the titles and abstracts of the studies to assess their eligibility criteria. In cases of disagreement between the two authors, a third author was consulted (T.D.). The full text of selected articles retrieved. The full text of these selected articles was reviewed by two independent authors to finalize eligibility criteria (B.T.G. and M.M.Ö.). Any discrepancies in full-text review were resolved through discussion involving all three authors (B.T.G. and M.M.Ö., and T.D.).

### Quality assessment

The quality of selected studies was assessed using the “Joanna Briggs Institute (JBI) Checklist for Prevalence Studies”. This tool evaluates various aspects of study methodology, including sampling strategies, data collection methods and analytical techniques. Each of the nine checklist items was scored “0” or “1”, resulting in a total score ranging from 0 to 9, where higher scores represent a lower risk of bias [21]. In our study, articles scoring above 6 on the JBI Checklist were considered to have a low risk of bias.

### Data extraction

Data extraction was performed independently by the two authors (B.T.G. and M.M.Ö.). The extracted information included first author’s name, publication year, survey period, sample size, data collection method, sample characteristics (gender, age, specific features, location), and vaccine hesitancy, which was then transferred to Microsoft Excel. Any disagreements between the authors were resolved by the third researcher (T.D.).

### Statistical procedure

Our data analysis was performed using Rev-Man 5 meta-analysis software. Pooled estimates of COVID-19 vaccine hesitancy were depicted using forest plots and a random-effects model. The Cohran Q test and *I*^2^ statistics were used to evaluate heterogeneity, which measures the proportion of the overall variance attributable to differences between studies rather than to chance. *I*^2^ values of 25%, 50%, and 75% are regarded as indicating low, moderate, and high heterogeneity, respectively. The generic inverse variance method was used in pooled prevalence data analysis (95% confidence interval [CI], random effect model with DerSimonian, and Laird method). Subgroup analyses were conducted based on participants (healthcare workers or not), conducted time of study (before or after national vaccination programme start in Turkey), and data collecting method (online or not). Publication bias was assessed using Egger’s test and a funnel plot. To address potential publication bias, we applied the trim and fill method using SPSS 29 (Statistical Package for Social Sciences).

## Results

### Study selection

**Figure 1.**
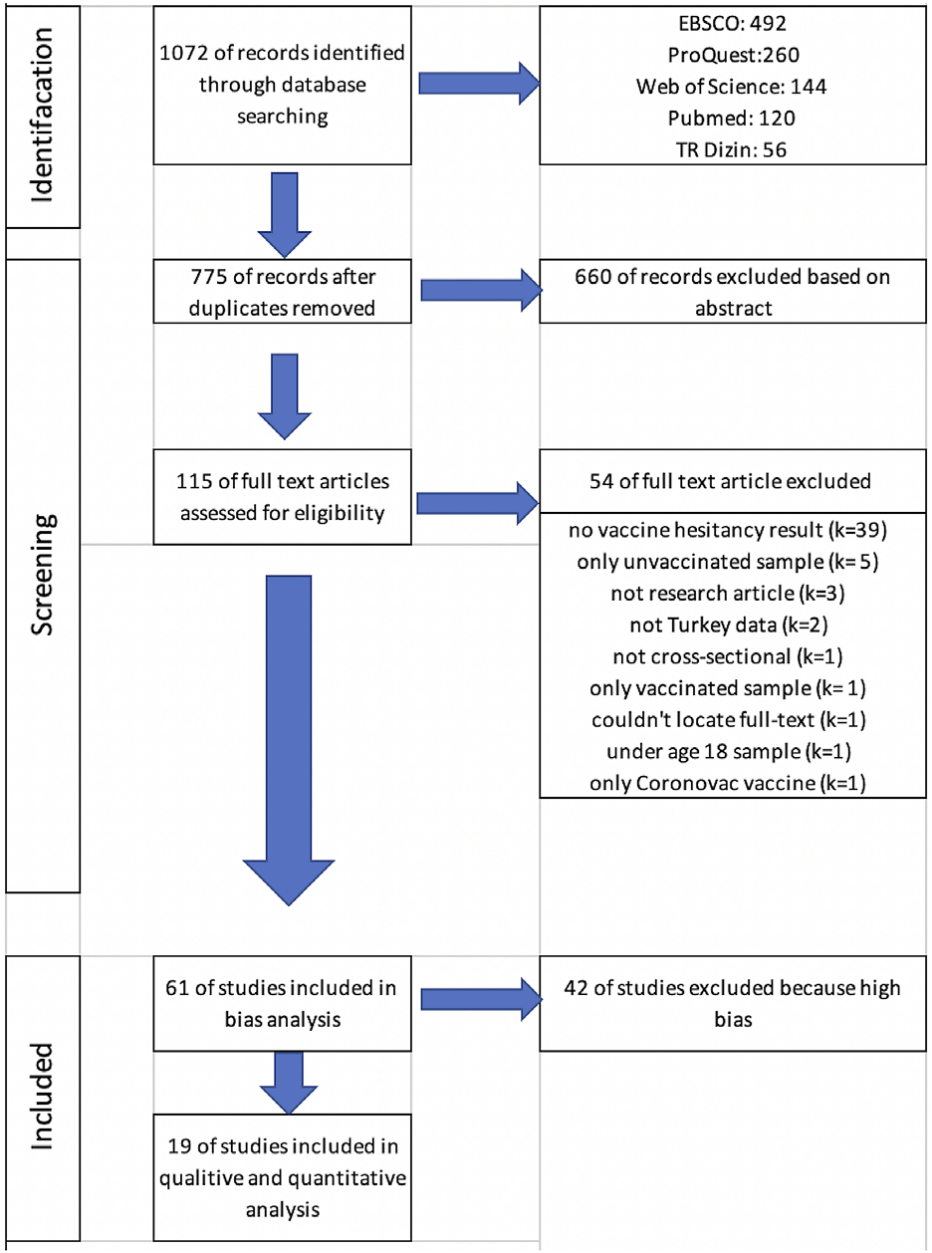
Flow-chart of bibliographic research.

A comprehensive search across databases initially yielded a total of 1,072 articles. After removing duplicate articles, 775 articles (297 duplicates) remained eligible. Subsequently, 660 articles were excluded following title and abstract screening. A full-text assessment of 115 articles led to the identification of 54 articles that did not meet the inclusion criteria (Supplementary Table S1). Following the bias assessment of the 61 articles that met the inclusion criteria, 19 of them had a low risk of bias and were included in the study. The article selection process is visually indicated in Figure 1, constructed in accordance with the PRISMA guidelines [19].

### Characteristics of the included studies

**Figure 2.**
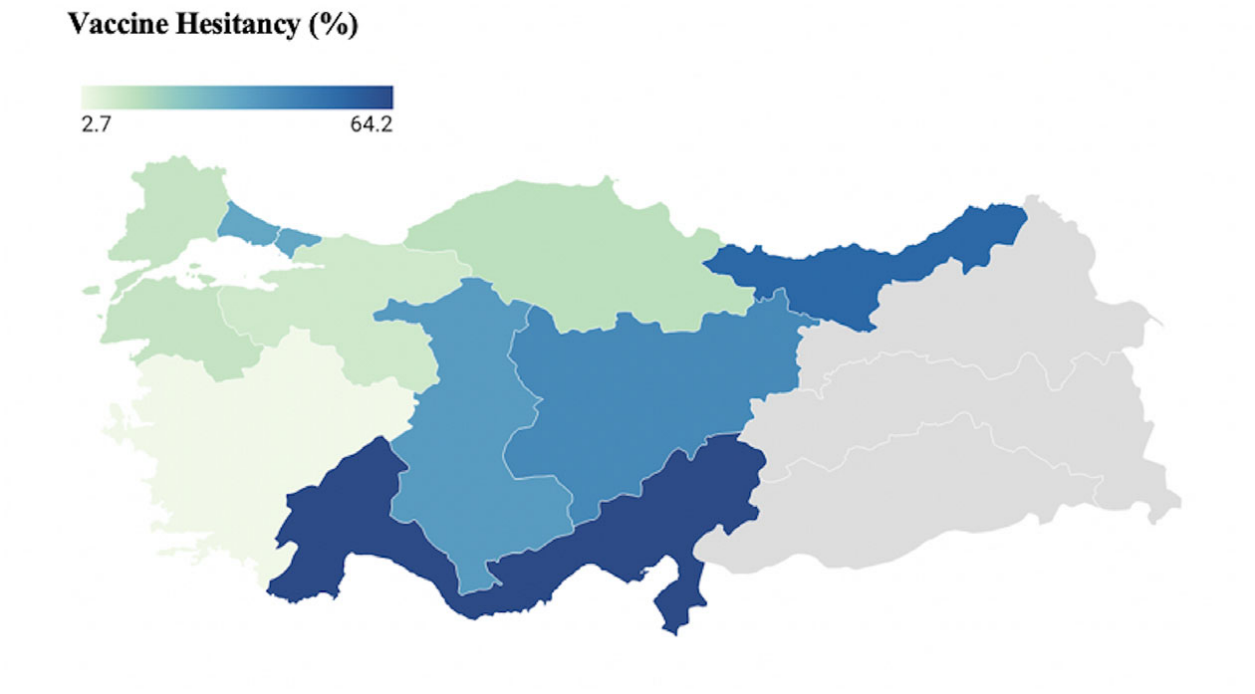
Prevalence of COVID-19 vaccine hesitancy according to the regions.

A total of 19 articles published between 2020 and 2023 were included in this systematic review and meta-analysis. The cumulative sample size across these articles encompassed 15,164 participants, with the study size ranging from 67 participants to 4,910 participants. Four of the studies represented Turkey. Other local studies were primarily conducted in Istanbul (n = 3), which is the most populous city of Turkey, accounting for 18.7% of the country population [22]. Additionally, studies representing various regions of Turkey were also included in the analysis (Figure 2). A predominant portion of the studies (n = 9, 47.4%) employed online data collection methods. Notably, a substantial proportion of studies (n = 12, 63.2%) focused on healthcare professionals or healthcare students. Out of 12 studies conducted in healthcare workers, 10 were conducted before the vaccination began, while among the seven studies not involving healthcare workers, six were conducted after the vaccination had started. A comprehensive summary of the study characteristics is available in Table 1.

**Table 1.** Characteristics of the selected studies in meta-analysis and systematic review

Author, year	Conduction time	N. of participant	Age^a^	Sex (male %)	Population	Location	Data collection method	Vaccine hesitancy (%)	Bias tool results
Gokdemir, 2022 [17]	April–June 21′	1,238	19.9 ± 1.9	30.9	Students studying in the field of health	Izmir-Balıkesir	Online	2.7	7/9
Pala, 2022 [28]	September 21’	1845	40.7 ± 0.4	71	Public institution employees	Bursa	Collected anonymously	9.6	8/9
Konus, 2022 [29]	November 21′	138	18.7 ± 0.8	44.2	Medical students (1st year)	Canakkale	Online	11.6	9/9
Erdem, 2021 [30]	May–June 21′	300	55.2 ± 12.9	35	Cancer patients	Samsun	Face to face; telephone	13.3	9/9
Alicilar, 2022 [31]	April 21’	336	21.1 ± 2.1	48.2	Medical students (3rd year)	Ankara	Online	14.3	8/9
Kizilkaya, 2023 [32]	August–September 21’	507	35 (19–54)	13.4	BMI > 30 patients	Istanbul	Face to face	15.4	8/9
Oygar, 2022 [33]	May–June 20’	4,910	<40 (70.7%)	27.3	HCWs working with paediatric patients	Turkey	Collected anonymously	19	7/9
Acar, 2021 [34]	December 20′–January 21’	494	31.7 ± 9.6	44.3	Family physicians	Turkey	Online	19.5	7/9
Okuyan, 2021 [35]	December 20′–January 21’	961	41.3 ± 11.7	32.5	Pharmacists	Turkey	Online	25.3	7/9
Lazarus, 2023 [36]	July 22′	1,000	18–29 (22%)	49.3	Not specific group	Turkey	Online	28	7/9
Ekmez, 2022 [37]	October 20′–March 21′	600	25.2 ± 9.4	0	Pregnant women	Istanbul	Face to face	35	8/9
Kaya, 2021 [38]	February 20′	739	21.2 ± 2.7	42.2	Medical students	Elazıg	Online	39.9	8/9
Akbulut, 2022 [39]	October–November 21’	460	30 (19–59)	70	Janitors and cleaning stuff	Malatya	Face to face	42.9	7/9
Yigit, 2021 [40]	December 20′	343	31.5 ± 7.9	23.1	HCWs working paediatric hospital	Ankara	Online	47.8	7/9
Akbulut, 2022 [41]	October–November 21′	161	37 (10)	41	Medical Secretary	Malatya	Face to face	49.1	7/9
Yarimoglu, 2021 [42]	January 21’	77	20–29 (49.4%)	36.4	Intensive care workers	Karaman	Face to face	49.4	7/9
Ikiışık, 2021 [43]	December 20′	276	38.6 ± 10.3	17.4	Family physicians, nurses	Istanbul	Online	49.7	8/9
Çatiker, 2022 [44]	January 21′	245	37.3 ± 9.1	21.2	Nurses	Ordu	Face to face	54.3	8/9
Kavuncuglu, 2021 [45]	January 20′	67	40 (26–62)	40.3	Family physicians, nurses	Hatay	Face to face	64.2	7/9

a Mean ± standard deviation (if available), or median (IQR) or highest group (percentage) are given.

### Vaccine hesitancy and subgroup analysis

**Figure 3.**
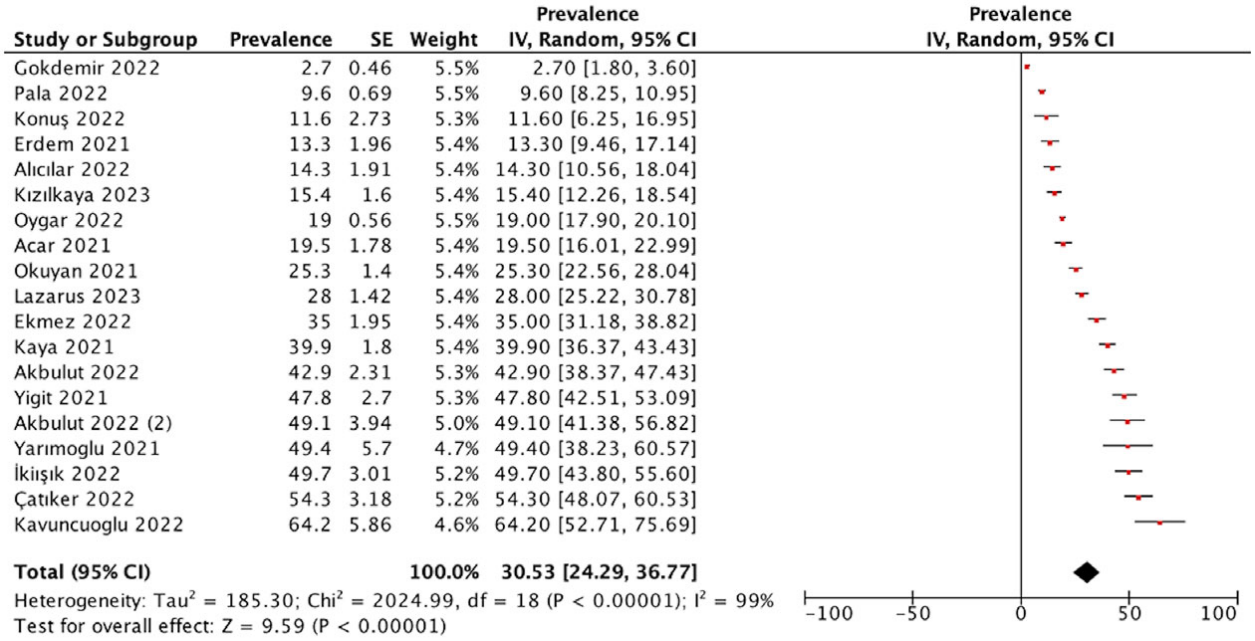
Forest plot of the pooled prevalence of vaccine hesitancy.

Through our meta-analysis, the pooled prevalence of COVID-19 vaccine hesitancy in Turkey was estimated to be 30.5% (95% Cl: 24.3–36.8, *I*^2^ = 99%, and *p* < 0.00001) (Figure 3).

**Figure 4.**
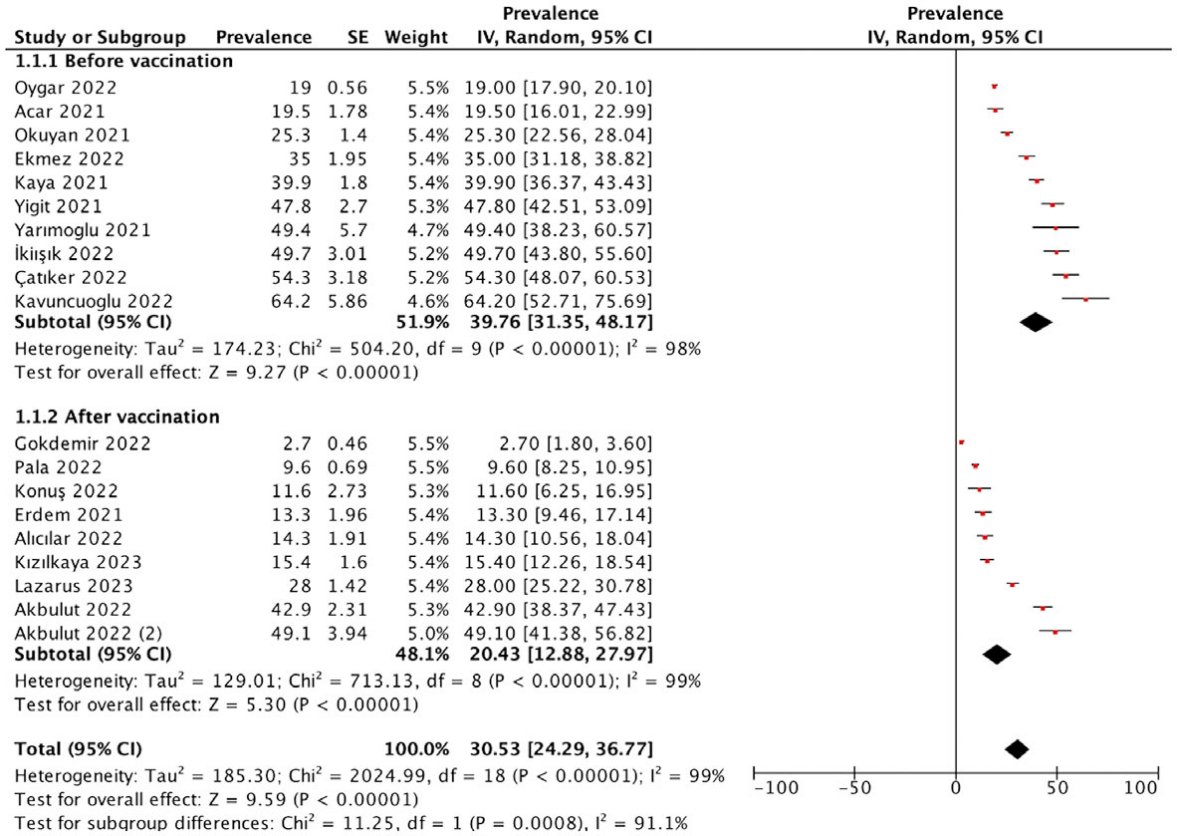
Forest plot of the pooled prevalence of vaccine hesitancy as determined by the different timing of survey (before vs. after vaccination start).

Before the initiation of national COVID-19 vaccination programme in Turkey, prior to 14 January 2021, the prevalence of vaccine hesitancy was 39.8% (95% Cl: 31.4–48.2%, *I*^2^ = 98%, and *p* < 0.00001). On the other hand, after the national COVID-19 vaccination programme, the prevalence of vaccine hesitancy was found to be 20.4% (95% Cl: 12.9–28.0%, *I*^2^ = 99%, and *p* < 0.00001) (Figure 4).

Among the studies conducted in healthcare workers and students in the healthcare domain, the prevalence of vaccine hesitancy was reported as 32.6% (95% Cl: 23.8–41.4%, *I*^2^ = 99%, and *p* < 0.00001). In contrast, studies encompassing other population groups reported a prevalence of vaccine hesitancy of 27.4% (95% Cl: 17.2–37.6%, *I*^2^ = 99%, and *p* < 0.00001).

Studies employing online data collections method yielded a prevalence of vaccine hesitancy of 26.5% (95% Cl: 14.6–38.3%, *I*^2^ = 99%, and *p* < 0.00001). Studies employing alternative data collection approaches, such as face-to-face or telephone interviews, reported a prevalence of vaccine hesitancy of 34.2% (95% Cl: 26.5–41.8%, *I*^2^ = 99%, and *p* < 0.00001).

Sensitivity analysis was conducted by removing studies one by one, and upon repeating the analysis, the pooled vaccine hesitancy was observed to be between 32.0% (95% CI: 26.3–37.7%, *I*^2^ = 98%, and *p* < 0.00001) and 28.9% (95% CI: 22.6–35.2%, *I*^2^ = 98%, and *p* < 0.00001).

### Risk of bias

Assessment of the 61 articles meeting the inclusion criteria was performed using the JBI Checklist for Prevalence Studies. Articles scoring six or below were excluded from the study. Upon examining the 42 articles, they were excluded due to high or moderate risk of bias. Excluded articles showed limitations in identifying the target groups, selecting an appropriate sampling method, reporting selected people’s access rates, and providing data the unreachable groups (Supplementary Table S2).

**Figure 5.**
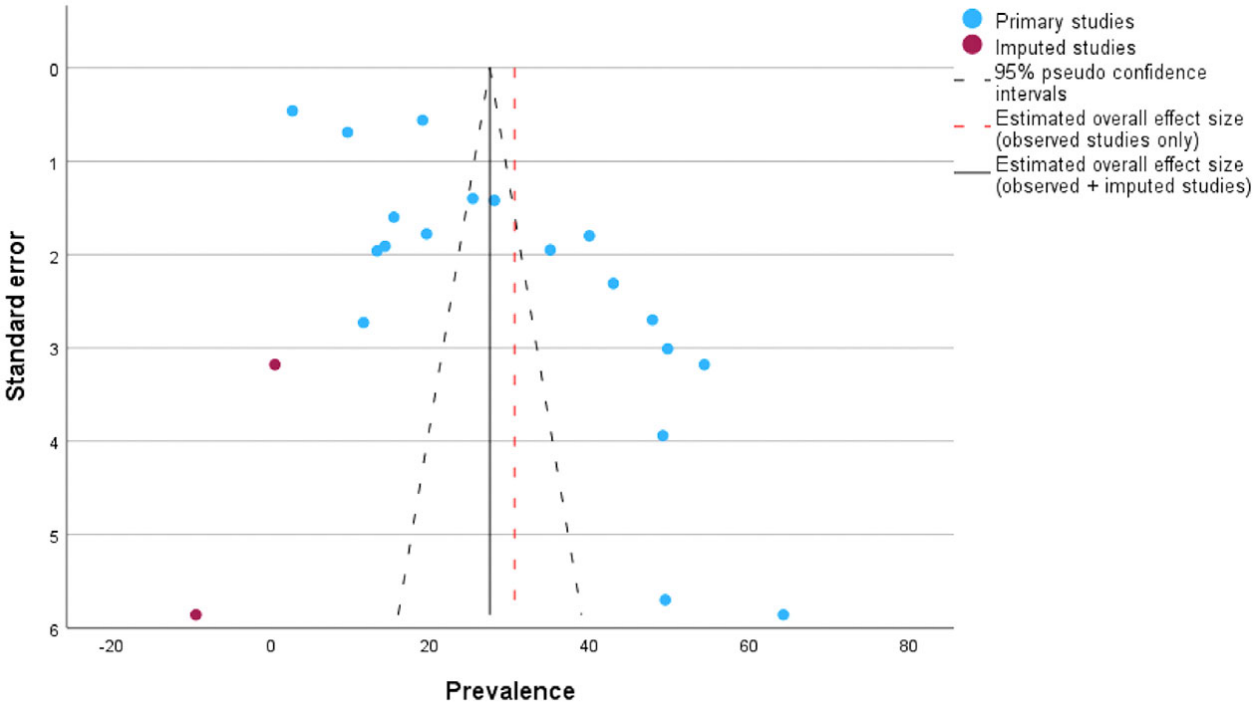
Funnel plot of included and imputed studies in the meta-analysis for publication bias.

It is observed that there are two studies with high prevalence and high standard error showing asymmetry in the funnel plot. Egger’s test also showed significant publication bias (*p* < 0.001). When the trim and fill method was applied, adjusting for the studies, the estimated prevalence of COVID-19 vaccine hesitancy was found to be 27.4% (95% Cl: 21.5–33.4). The adjusted funnel plot for publication bias is presented in Figure 5.

## Discussion

Vaccine hesitancy is a significant public health problem worldwide. In a Canadian meta-analysis, COVID-19 vaccine hesitancy was reported as 42.3% [11]. A meta-analysis covering 33 studies from low- and middle-income countries found vaccine hesitancy to be 38.2% [16]. In the United States, the willingness to get vaccinated for COVID-19 was reported 61% [23]. COVID-19 vaccine acceptance were reported at 39.9% in Jordan [13], 48.9% in Africa [24], ranging from 51.6% to 58.7% in Ethiopia according to various meta-analysis studies [14, 25, 26]. COVID-19 vaccine acceptance was shown to be lowest in the Middle East at 46% and the highest in Asia at 75% [27]. According to presented meta-analysis study of Turkey, the pooled prevalence of the COVID-19 vaccine hesitancy was 30.5%.

The relatively lower vaccine hesitancy observed in our study may be attributed two factors. Firstly, a considerable portion of studies in Turkey were conducted after the commencement of COVID-19 vaccination, while most other meta-analyses primarily focused on studies conducted at the beginning of the COVID-19 pandemic. For instance, in Alimohamadi et al.’s meta-analysis, 73 articles were reviewed from different countries. Out of these studies, 67 (90%) were conducted in 2020, with the remaining conducted in 2021 [27]. Among the 19 studies included in the meta-analysis conducted in Canada, five of them (26.3%) were conducted after 2020 [11]. The subgroup analysis of our study revealed that the vaccine hesitancy was 39.8% before January 2021, whereas this was decreased to 20.4% after January 2021. The decrease may be attributed to regulatory approvals, enhanced scientific literature data, the success stories from other countries, and the positive outcomes of vaccinated individuals.

Secondly, relatively low vaccine hesitancy could be associated with the high numbers of healthcare workers and students included in our study. In a meta-analysis of 14 studies among healthcare workers in Italy, the prevalence of COVID-19 vaccine hesitancy was found to be 13.1% [12]. In our study, the COVID-19 vaccine hesitancy was found to be 32.6% in studies conducted among healthcare workers, while in other studies, it was 27.4%. The reason for the relatively high prevalence of vaccine hesitancy in our study among healthcare workers may be that most of the studies conducted with healthcare workers were conducted before vaccination program began (83.3%), while most of the other studies were conducted afterward (85.7%). Therefore, the high vaccine hesitancy observed in studies conducted before the vaccination program might have contributed to the high hesitancy among healthcare workers.

In Turkey, although COVID-19 vaccine hesitancy has been found to be relatively low for the reasons we have mentioned, it can still be a significant obstacle to achieving the WHO’s goals of vaccinating 70% of the population and 100% of healthcare workers and vulnerable groups. We suggested that early initiation of vaccination programmes contributed to reducing the vaccine hesitancy in society and played a crucial role in the management of pandemics.

There are some limitations to our study. Firstly, a notable portion of studies (*n* = 42, 68.9%) identified in this systematic review was excluded due to high bias (Supplementary Table S2). These excluded articles exhibited significant methodological shortcomings in identifying the target population, selecting samples, and reporting the rate of accessibility to the target group. We emphasize the need for studies with stronger methodologies and less bias in investigating vaccine hesitancy. Secondly, the majority of the studies (*n* = 15) included in our analysis focused on specific cities or regions within Turkey, revealing varying levels of vaccine hesitancy (Figure 2). Given the influence of cultural and socioeconomic factors on hesitancy, it is essential to conduct more studies across diverse regions to comprehensively understand the extent of this phenomenon. Thirdly, publication bias was detected in our study. Therefore, trim and fill method was applied, and the adjusted vaccine hesitancy was calculated to be 27.4%. Fourthly, high heterogeneity was observed in our analyses. Subgroup analyses based on study time, study group, and data collection method also revealed high heterogeneity. The study possesses notable strengths. Firstly, it employed a rigorous search strategy across multiple databases, ensuring a comprehensive inclusion of relevant articles. Additionally, it is the first meta-analysis conducted in Turkey specifically addressing COVID-19 vaccine hesitancy. The study is characterized by a substantial sample size and exhibits strong methodological rigor.

## Conclusion

In our study, COVID-19 vaccine hesitancy in Turkey was found to be 30.5% (95% Cl: 24.3–36.8). There is a significant difference in vaccine hesitancy between studies conducted before the vaccination programme started and those conducted after the initiation of vaccination (39.8% vs. 20.4, respectively). The findings emphasize the significance of timely vaccination programmes and the necessity of the studies with strong methodologies to effectively combat hesitancy. Since vaccine hesitancy vary across regions, comprehensive research evaluating demographic and epidemiological data is essential for developing targeted interventions and fostering vaccine acceptance.

## Supporting information

Gulle et al. supplementary material Gulle et al. supplementary material

## Data Availability

The data related to the study are provided in the supplementary materials. For more information, you can request from the corresponding author.

## Supplementary material

For supplementary material accompanying this paper visit http://doi.org/10.1017/S0950268823001875. click here to view supplementary material
