# Supplementary material for: COVID-19 vaccine hesitancy in Turkey: A systematic review and meta-analysis
Source: Epidemiol Infect. 2023 Nov 24;151:e199. doi: 10.1017/S0950268823001875 (PMC10728987; doi:10.1017/S0950268823001875)
Supplement: Gulle et al. supplementary material [file S0950268823001875sup001.docx]

**Epidemiology and Infection**

**COVID-19 Vaccine Hesitancy in Turkey: A Systematic Review and Meta-analysis**

Bugra Taygun GULLE, Meryem Merve ONER, Tuba DAL

**Supplementary Material**

**Supplementary Table 1. Articles excluded after full-text assessment and the reason for their exclusion**

|  |  |  |  | |  |  |  | |  |  | |  |  |  |  |
| --- | --- | --- | --- | --- | --- | --- | --- | --- | --- | --- | --- | --- | --- | --- | --- |
| **Name of Study** | | | | **Publication year** | | | | **First author** | | | **Reason of Exclusion** | | | |  |
| A Community-based Study in the Central District of Giresun: COVID-19 Vaccine Hesitancy | | | | 2022 | | | | Teneler AA | | | Only unvaccinated sample | | | |  |
| A Cross-sectional Study Evaluating COVID-19 Vaccine Literacy: The Example of Antalya Province | | | | 2022 | | | | Acar AB | | | No vaccine hesitancy result | | | |  |
| A Mixed Methods Study of Health Care Professionals' Attitudes Towards Vaccination in 15 Countries | | | | 2022 | | | | Alasmari A | | | No vaccine hesitancy result | | | |  |
| Acceptability of Covid-19 Vaccine and Factors Affecting Vaccine Hesitation in Pregnant Health Care Workers | | | | 2022 | | | | Tokalıoglu EO | | | Only unvaccinated sample | | | |  |
| Attitudes and Opinions of Medical Students and Health Care Professionals About the Covid-19 Vaccine (a Quantitative Study from a Medical School and Its Affiliated Hospitals) | | | | 2021 | | | | Ulman YI | | | Not research article | | | |  |
| Attitudes and Practices Against COVID-19 Vaccines in Turkiye | | | | 2022 | | | | Atac O | | | Only unvaccinated sample | | | |  |
| Attitudes of Family Medicine Residents in Ankara Towards COVID-19 Vaccines Administered in Turkey: a Descriptive, Cross-sectional Study | | | | 2022 | | | | Cevik HS | | | No vaccine hesitancy result | | | |  |
| Attitudes Toward COVID-19 Vaccine: Investigating the Role of COVID-19 Related Factors and Trait Personality | | | | 2022 | | | | Deniz ME | | | No vaccine hesitancy result | | | |  |
| Attitudes Towards COVID-19 Vaccination, Vaccine Hesitancy and Vaccine Literacy Among Unvaccinated Young Adults | | | | 2022 | | | | Yılmazel G | | | No vaccine hesitancy result | | | |  |
| Birinci Basamak Saglik Calisanlarinin Koronavirus Salgini Surecinde Covid-19 Asisina Yonelik Tutumlari ve Covid-19 Hastalik Algilari | | | | 2022 | | | | Ozkan F | | | No vaccine hesitancy result | | | |  |
| Can We Persuade Those Who Hesitate to Get the COVID-19 Vaccine? | | | | 2022 | | | | Gamsizkan Z | | | Not research article | | | |  |
| Correlations Between Attitudes Towards the COVID-19 Vaccine and Psychiatric Symptoms Among Pregnant Women | | | | 2023 | | | | Ayık B | | | No vaccine hesitancy result | | | |  |
| COVID-19 Infection, Vaccine Status, and Avoidance Behaviors in Adults with Attention Deficit and Hyperactivity Disorder: A Cross-sectional Study | | | | 2022 | | | | Kilic O | | | No vaccine hesitancy result | | | |  |
| COVID-19 Oykusu Koronafobi ve Asi Tutumlarinda Belirleyici Olabilir mi? | | | | 2022 | | | | Ozturk R | | | No vaccine hesitancy result | | | |  |
| COVID-19 Vaccination Rates and Factors Affecting Vaccine Hesitancy among Pregnant Women during the Pandemic Period in Turkey: A Single-Center Experience | | | | 2022 | | | | Ozen DSK | | | No vaccine hesitancy result | | | |  |
| COVID-19 Vaccination-Related Thoughts, Behaviors, and Expectations of the Academic Personnel in Hacettepe University Medical School | | | | 2022 | | | | Kara SA | | | Only Coronovac vaccine | | | |  |
| COVID-19 Vaccine Acceptance Among Primary Care Residents in Middle-Black Sea Region of Turkey | | | | 2021 | | | | Yilmazel G | | | Couldn't locate full-text | | | |  |
| COVID-19 Vaccine Acceptance is Associated with Vaccine Hesitancy, Perceived Risk and Previous Vaccination Experiences | | | | 2021 | | | | Dolu I | | | No vaccine hesitancy result | | | |  |
| COVID-19 Vaccine Hesitancy and Related Factors Among Unvaccinated Pregnant Women During the Pandemic Period in Turkey | | | | 2023 | | | | Sezerol MA | | | No vaccine hesitancy result | | | |  |
| COVID-19 Vaccine Hesitancy in Healthcare Personnel: A University Hospital Experience | | | | 2021 | | | | Esen BK | | | No vaccine hesitancy result | | | |  |
| COVID-19 Vaccine Hesitancy is Associated with Beliefs on the Origin of the Novel Coronavirus in the UK and Turkey | | | | 2020 | | | | Salali GD | | | Not research article | | | |  |
| Determining the Relationship Between Coronavirus Anxiety and Attitudes Towards the COVID-19 Vaccination in Students from Vocational School of Health Services | | | | 2022 | | | | Parlak AG | | | No vaccine hesitancy result | | | |  |
| Digital Health Literacy for COVID-19 Vaccination and Intention to be Immunized: A Cross Sectional Multi-country Study Among the General Adult Population | | | | 2022 | | | | Marzo RR | | | No vaccine hesitancy result | | | |  |
| Investigation of Dentists' Willingness to have COVID-19 Vaccine | | | | 2022 | | | | Gumustas B | | | No vaccine hesitancy result | | | |  |
| Do Healthy Lifestyle Behaviors Affect COVID-19 Vaccination Attitudes in Generation Z? | | | | 2022 | | | | Barut S | | | No vaccine hesitancy result | | | |  |
| Experience and Opinions of Healthcare Professionals on COVID-19 and Inactive COVID-19 Vaccine (Coronavac, Developed by Sinovac of China) | | | | 2022 | | | | Tetik BK | | | No vaccine hesitancy result | | | |  |
| Factors Affecting Negative Attitudes Towards COVID-19 Vaccines | | | | 2023 | | | | Bozkurt V | | | No vaccine hesitancy result | | | |  |
| Global Prevalence and Drivers of Dental Students’ COVID-19 Vaccine Hesitancy | | | | 2021 | | | | Riad A | | | No vaccine hesitancy result | | | |  |
| In or Out? Identifying The Factors Playing Role In Covid-19 Vaccine Decision in Turkey | | | | 2023 | | | | Ankara HG | | | No vaccine hesitancy result | | | |  |
| Investigation Of Reasons For the COVID-19 Vaccine Hestiancy of CIitizens Over the Age of 18 | | | | 2022 | | | | Tekin HH | | | No vaccine hesitancy result | | | |  |
| Investigation of Perceived Fear of COVID-19 and Vaccine Hesitancy in Nursing Students | | | | 2021 | | | | Yeşiltepe A | | | No vaccine hesitancy result | | | |  |
| Is Vaccine Hesitancy Affected by Distrust in the Healthcare System? A Study in Turkish Population | | | | 2023 | | | | Ozer O | | | No vaccine hesitancy result | | | |  |
| Knowledge, Attitudes, and Perception Towards COVID-19 Vaccination Among the Adult Population: A Cross-Sectional Study in Turkey | | | | 2022 | | | | Sonmezer MC | | | No vaccine hesitancy result | | | |  |
| Linking the Behavioral Immune System to COVID-19 Vaccination Intention: The Mediating Role of the Need for Cognitive Closure and Vaccine Hesitancy | | | | 2022 | | | | Solak C | | | No vaccine hesitancy result | | | |  |
| Neden Herkes Asi Olmuyor? Covid-19 Asi Tutumu ile Iliskili Degiskenler | | | | 2022 | | | | Basal H | | | No vaccine hesitancy result | | | |  |
| Olum Belirginliginin ve Kovid-19 Korkusunun Kovid-19 Asisi Karsitligina Etkisi: Yari Deneysel Bir Calisma | | | | 2022 | | | | Harlak H | | | No vaccine hesitancy result | | | |  |
| Opinions on COVID-19 Vaccines, Professional Continuity and Protective Practices During the Pandemic: A Descriptive Study on Turkish Dentists | | | | 2022 | | | | Ozler CO | | | No vaccine hesitancy result | | | |  |
| Peoples' Attitude Toward COVID-19 Vaccine, Acceptance, and Social Trust Among African and Middle East countries | | | | 2021 | | | | Faezi NA | | | Not Turkey | | | |  |
| Perception Towards Vaccine Effectiveness in Controlling COVID-19 Spread in Rural and Urban Communities: A Global Survey | | | | 2022 | | | | Marzo RR | | | No vaccine hesitancy result | | | |  |
| Perspectives of Dermatology Specialists and Residents on COVID-19 Vaccines: A Questionnaire-based Survey | | | | 2021 | | | | Tanacan E | | | No vaccine hesitancy result | | | |  |
| Status of COVID-19 Infection and Vaccination in People Aged 18 Years and over in a Town: A Cross-Sectional Study in Turkey | | | | 2022 | | | | Medeni V | | | No vaccine hesitancy result | | | |  |
| The Attitudes of Healthcare Professionals in Turkey Toward the Coronavirus Vaccine | | | | 2022 | | | | Azizoglu F | | | No vaccine hesitancy result | | | |  |
| The COVID-19 Vaccination Acceptance/Hesitancy Rate and Its Determinants Among Healthcare Workers of 91 Countries: A multicenter Cross-sectional Study | | | | 2022 | | | | Askarian M | | | Not Turkey | | | |  |
| The Effect of the COVID-19 Pandemic on Vaccination Behaviour of Individuals over the Age of 65 Years in Turkey: Single-Centre Experience | | | | 2022 | | | | Ergin AU | | | Only vaccinated sample | | | |  |
| The Mediating Role of Health Literacy on the Relationship Between Health Care System Distrust and Vaccine Hesitancy During COVID-19 Pandemic | | | | 2022 | | | | Turhan Z | | | No vaccine hesitancy result | | | |  |
| The Percentage of Hesitation and Factors associated with Acceptance or Refusal for COVID-19 Vaccine: Does Training About Vaccines by Allergist Affect Personal Decision? | | | | 2023 | | | | Sayaca N | | | Not cross sectional | | | |  |
| The Relationship Between COVID-19 Awareness and Vaccinne Hesitancy Among University Students | | | | 2022 | | | | Eren H | | | No vaccine hesitancy result | | | |  |
| The Roles of National and Global Identities and Leaders in the Acceptance of COVID-19 Vaccines Developed by Different Countries | | | | 2022 | | | | Akfirat S | | | No vaccine hesitancy result | | | |  |
| Turkish Healthcare Workers' Personal and Parental Attitudes to COVID-19 Vaccination From a Role Modeling Perspective | | | | 2022 | | | | Oncel S | | | No vaccine hesitancy result | | | |  |
| Turkiye’de Insanlarin COVID-19 Asisina Bakisi | | | | 2021 | | | | Yılmaz HI | | | Under 18 age sample | | | |  |
| Ucuncu Basamak Bir Hastanede Saglik Calisanlarinin COVID-19 Enfeksiyonu ve Asilamasi Hakkindaki Yaklasim ve Davranislari | | | | 2022 | | | | Salman Z | | | No vaccine hesitancy result | | | |  |
| Understanding Vaccine Hesitancy: Social Isolation in Relation to Social Media Addiction and COVID-19 Anxiety | | | | 2022 | | | | Erinc ZO | | | No vaccine hesitancy result | | | |  |
| Vaccine Hesitancy Among Healthcare Professionals and the General Population: Second Important Step in the Fight Against COVID-19 | | | | 2023 | | | | Metin S | | | Only unvaccinated sample | | | |  |
| Knowledge and Attitudes of Pregnant Women about Coronavirus Vaccines in Turkiye | | | | 2022 | | | | Odabas RK | | | Only unvaccinated sample | | | |  |

| **Supplementary Table 2. The results of bias analysis for articles remaining after full text assessment** | | | | | | | | | | | | |
| --- | --- | --- | --- | --- | --- | --- | --- | --- | --- | --- | --- | --- |
| Name of study | Publication year | First author | Q1 | Q2 | Q3 | Q4 | Q5 | Q6 | Q7 | Q8 | Q9 | Total points |
| Impact of Corona-phobia on Attitudes and Acceptance Towards COVID-19 Vaccine Among Cancer Patients: a Single-center Study | 2021 | Erdem D | 1 | 1 | 1 | 1 | 1 | 1 | 1 | 1 | 1 | 9 |
| Tıp Fakültesi Birinci Sınıf Öğrencilerinde Aşı Kararsızlığı ve COVID-19 Aşısı Olma Durumu | 2022 | Konuş S | 1 | 1 | 1 | 1 | 1 | 1 | 1 | 1 | 1 | 9 |
| Barriers to Coronavirus Disease 19 Vaccination in Patients with Obesity | 2023 | Kızılkaya MC | 0 | 1 | 1 | 1 | 1 | 1 | 1 | 1 | 1 | 8 |
| Acceptability of a COVID-19 Vaccine and Role of Knowledge, Attitudes and Beliefs on Vaccination Willingness Among Medical Students | 2021 | Kaya M.O | 1 | 1 | 1 | 1 | 0 | 1 | 1 | 1 | 1 | 8 |
| Ankara Üniversitesi Tıp Fakültesi Dönem 3 Öğrencilerinin COVID-19 Aşılarına Karşı Tutumları ve İlişkili Faktörler | 2022 | Alıcılar HE | 1 | 1 | 1 | 1 | 0 | 1 | 1 | 1 | 1 | 8 |
| Assessment of Factors Affecting Attitudes and Knowledge of Pregnant Women about COVID-19 Vaccination | 2022 | Ekmez M | 1 | 1 | 1 | 1 | 0 | 1 | 1 | 1 | 1 | 8 |
| Bir Kamu Kurumu Calışanlarinda COVID-19 Asi Tutumu ve Iliskili Etmenler | 2022 | Pala K | 1 | 1 | 1 | 1 | 0 | 1 | 1 | 1 | 1 | 8 |
| COVID-19 Vaccine Hesitancy and Related Factors Among Primary Healthcare Workers in a District of Istanbul: a Cross-sectional Study from Turkey | 2021 | İkiışık, H | 1 | 1 | 1 | 1 | 0 | 1 | 1 | 1 | 1 | 8 |
| Hemsirelerde COVID-19 Asisiniin Kabulu ve Kararsizligi | 2022 | Çatıker A | 1 | 1 | 1 | 1 | 0 | 1 | 1 | 1 | 1 | 8 |
| A Survey of COVID-19 Vaccine Acceptance Across 23 Countries in 2022 | 2023 | Lazarus JV | 1 | 1 | 1 | 1 | 0 | 1 | 1 | 1 | 0 | 7 |
| An Analysis of the Attitudes of Family Physicians Towards the COVID-19 Vaccine | 2021 | Acar AB | 1 | 0 | 1 | 1 | 1 | 1 | 1 | 1 | 0 | 7 |
| Attitudes of Family Health Professionals on COVID-19 Vaccines and an Evaluation of Underlying Factors in Samandağ, Hatay, Turkey | 2021 | Kavuncuğlu D | 1 | 1 | 0 | 1 | 0 | 1 | 1 | 1 | 1 | 7 |
| Bir Egitim Arastirma Hastanesi Yogun Bakim Calisanlarinin COVID-19 Asilari Hakkindaki Tutumlari: Bir Tanimlayici Calisma | 2021 | Yarımoğlu R | 1 | 1 | 0 | 1 | 0 | 1 | 1 | 1 | 1 | 7 |
| Evaluation of COVID-19 Vaccine Acceptance of Healthcare Providers in a Tertiary Pediatric Hospital | 2021 | Yigit M | 1 | 1 | 1 | 1 | 0 | 1 | 1 | 1 | 0 | 7 |
| Evaluation of Vaccination Status of Health Care Workers for Recommended Vaccines and Their Acceptance of SARS-CoV-2 Vaccines | 2022 | Oygar PD | 1 | 1 | 1 | 1 | 0 | 1 | 1 | 1 | 0 | 7 |
| Evaluation of Vaccine Hesitancy and Anxiety Levels among Hospital Cleaning Staff and Caregivers during COVID-19 Pandemic | 2022 | Akbulut S | 1 | 0 | 1 | 1 | 0 | 1 | 1 | 1 | 1 | 7 |
| Evaluation of Vaccine Hesitancy, Anti-Vaccination, and Anxiety Levels for Medical Secretaries During COVID-19 Pandemic | 2022 | Akbulut S | 1 | 0 | 1 | 1 | 0 | 1 | 1 | 1 | 1 | 7 |
| Factors Associated with Turkish Pharmacists' Intention to Receive COVID-19 Vaccine: an Observational Study | 2021 | Okuyan B | 1 | 1 | 1 | 1 | 0 | 1 | 1 | 1 | 0 | 7 |
| Vaccine Hesitancy Among University Students of Healthcare | 2022 | Gökdemir Ö | 1 | 0 | 1 | 1 | 0 | 1 | 1 | 1 | 1 | 7 |
| Are Midwifery Students Ready for the COVID-19 Vaccine? The Decision to Vaccinate and Affecting Factors | 2021 | Özçoban F | 1 | 0 | 1 | 1 | 0 | 1 | 1 | 1 | 0 | 6 |
| COVID-19 Vaccine Hesitancy and Its Relationship With Illness Risk Perceptions, Affect, Worry, and Public Trust: An Online Serial Cross-Sectional Survey From Turkey | 2021 | Küçükkarapınar M | 1 | 0 | 1 | 1 | 0 | 1 | 1 | 1 | 0 | 6 |
| COVID‐19 Vaccine Hesitancy: A Community‐Based Research in Turkey | 2021 | İkiışık, H | 1 | 1 | 0 | 1 | 0 | 1 | 1 | 1 | 0 | 6 |
| Nursing Students’ Opinions About the COVID-19 Vaccine: A Descriptive, Cross-Sectional Study | 2022 | Köse S | 1 | 0 | 1 | 1 | 0 | 1 | 1 | 1 | 0 | 6 |
| Pediatricians' COVID-19 Experiences and Views on the Willingness to Receive COVID-19 Vaccines: a Cross-sectional Survey in Turkey | 2021 | Gönüllü E | 1 | 0 | 1 | 1 | 0 | 1 | 1 | 1 | 0 | 6 |
| Perceived COVID-19 Vaccine Effectiveness, Acceptance, and Drivers of Vaccination Decision-making Among the General Adult Population: A Global Survey of 20 countries | 2022 | Marzo RR | 0 | 0 | 1 | 1 | 1 | 1 | 1 | 1 | 0 | 6 |
| The Attitude of Turkish Physicians Toward COVID-19 Vaccination and the Effects of Vaccination on Their Mental Health | 2022 | Sancak B | 0 | 0 | 1 | 1 | 0 | 1 | 1 | 1 | 1 | 6 |
| The Attitude of Dentists Towards COVID-19 Vaccination in Turkey: A Survey Study | 2022 | Kaya DI | 0 | 0 | 1 | 1 | 1 | 1 | 1 | 1 | 0 | 6 |
| A Global Survey of COVID-19 Vaccine Acceptance Among Healthcare Workers | 2021 | Noushad M | 0 | 0 | 0 | 1 | 0 | 1 | 1 | 1 | 0 | 5 |
| Approaches of the Physicians on COVID-19 Vaccination: An Online Survey from Türkiye | 2022 | Doğan CS | 0 | 0 | 1 | 1 | 0 | 1 | 1 | 1 | 0 | 5 |
| Assesment of Physicians? Attitudes Towards COVID-19 Vaccine | 2022 | Muz FNO | 0 | 0 | 1 | 1 | 0 | 1 | 1 | 1 | 0 | 5 |
| Attitude Towards COVID-19 Vaaccine and Affecting Factors in Adults From Turkey | 2022 | Çolak M. | 0 | 0 | 1 | 1 | 0 | 1 | 1 | 1 | 0 | 5 |
| Bireylerin COVID-19 Asisi Hakkinda Dusunce ve Tutumlari Kesitsel Bir Calisma | 2022 | Çopur EÖ | 0 | 0 | 1 | 1 | 0 | 1 | 1 | 1 | 0 | 5 |
| COVID-19 Vaccine Acceptance in Pregnant Women | 2021 | Ayhan SG | 0 | 0 | 1 | 1 | 0 | 1 | 1 | 1 | 0 | 5 |
| COVID-19 Vaccine Hesitancy in Pregnancy: A Cross-Sectional Study | 2022 | Ercan A | 0 | 0 | 1 | 1 | 0 | 1 | 1 | 1 | 0 | 5 |
| COVID-19’a İlişkin Asi Karsitligina Etki Eden Faktörler: Türkiye'den Bir Online Anket Calismasi | 2022 | Mete B | 0 | 0 | 1 | 1 | 0 | 1 | 1 | 1 | 0 | 5 |
| Covid-19 Vaccination Acceptance and Hesitancy Among the Turkish Adult Population | 2021 | Köse S | 0 | 0 | 1 | 1 | 0 | 1 | 1 | 1 | 0 | 5 |
| Factors Affecting the Acceptability of COVID-19 Vaccine in the Postpartum Period | 2021 | Oluklu D | 0 | 0 | 1 | 1 | 0 | 1 | 1 | 1 | 0 | 5 |
| Hesitancy Towards a COVID-19 Vaccine Among Midwives in Turkey During the COVID-19 Pandemic: A Cross-sectional Web-based Survey | 2021 | Kaya L | 0 | 0 | 1 | 1 | 0 | 1 | 1 | 1 | 0 | 5 |
| Immunization in Health Employees: Relationship of Confidence and Attitude | 2021 | Yıldırım D | 0 | 0 | 1 | 1 | 0 | 1 | 1 | 1 | 0 | 5 |
| Investigation of the Relationships Between Perceived Causes of COVID-19, Attitudes Towards Vaccine and Level of Trust in Information Sources from the Perspective of Infodemic: the Case of Turkey | 2021 | Karabela ŞN | 0 | 0 | 1 | 1 | 0 | 1 | 1 | 1 | 0 | 5 |
| Investigation of Turkish Community Vaccine Hesitancy and Reasons During the COVID-19 Pandemic: A Descriptive Study | 2022 | Bayssal E | 0 | 0 | 1 | 1 | 0 | 1 | 1 | 1 | 0 | 5 |
| Psychological Factors Affecting COVID-19 Vaccine Hesitancy | 2022 | Nazlı ŞB | 0 | 0 | 1 | 1 | 0 | 1 | 1 | 1 | 0 | 5 |
| Revisiting COVID-19 Vaccine Hesitancy Around the World Using Data from 23 Countries in 2021 | 2022 | Lazarus J. V | 0 | 0 | 0 | 1 | 0 | 1 | 1 | 1 | 1 | 5 |
| The Attitudes of University Students Who Received Online Education During the Pandemic Towards COVID-19 Vaccines | 2021 | Şahin NT | 0 | 0 | 1 | 1 | 0 | 1 | 1 | 1 | 0 | 5 |
| The Evaluation of Vaccine Hesitancy and Refusal for Childhood Vaccines and the COVID-19 Vaccine in Individuals Aged Between 18 and 25 Years | 2021 | Soysal G | 0 | 0 | 1 | 1 | 0 | 1 | 1 | 0 | 1 | 5 |
| The Willingness to Accept the COVID-19 Vaccine and Affecting Factors Among Healthcare Professionals: A Cross-sectional Study in Turkey | 2021 | Kaplan K | 0 | 0 | 1 | 1 | 0 | 1 | 1 | 1 | 0 | 5 |
| Turkiye'de Tip Fakultesi Ogrencilerinin Asi, Bagisiklama, Asi Kararsizligi ve COVID-19 Asisi Hakkindaki Bilgi ve Dusunceleri | 2021 | Özbalıkçı E | 0 | 0 | 1 | 1 | 0 | 1 | 1 | 1 | 0 | 5 |
| Vaccine Hesitancy of the COVID-19 by Health Care Personnel | 2020 | Köse S | 0 | 0 | 1 | 1 | 0 | 1 | 1 | 1 | 0 | 5 |
| What Do Health Care Professionals Think About Covid-19 Vaccine Applications: A University Example | 2021 | Kurtuluş Ş | 0 | 0 | 1 | 1 | 0 | 1 | 1 | 1 | 0 | 5 |
| While Studies on COVID-19 Vaccine is Ongoing, the Public's Thoughts and Attitudes to the Future COVID-19 Vaccine | 2020 | Akarsu B | 0 | 0 | 1 | 1 | 0 | 1 | 1 | 1 | 0 | 5 |
| A Survey Study of COVID-19 Vaccine Hesitancy of Relatives of Patiennts Admitted to a Training and Research Hospital in Istanbul | 2022 | Özkaya H | 0 | 0 | 1 | 1 | 1 | 1 | 1 | 0 | 0 | 5 |
| Willingness to Get the COVID-19 Vaccine Among Patients with Rheumatic Diseases, Healthcare Workers and General Population in Turkey: a Web-based Survey | 2021 | Yurttaş B | 0 | 0 | 1 | 1 | 0 | 1 | 1 | 1 | 0 | 5 |
| Bir Aile Sagligi Merkezine Basvuran Hastalarin Covid-19 Asisina Yonelik Dusunceleri Ile Covid-19 Korkusunun Degerlendirilmesi | 2022 | Güngör S | 0 | 0 | 0 | 1 | 0 | 1 | 1 | 1 | 0 | 4 |
| COVID-19 and Vaccine Hesitancy: Could Health Literacy be the Solution? | 2022 | Kocaay F | 0 | 0 | 0 | 1 | 0 | 1 | 1 | 1 | 0 | 4 |
| Determinants of COVID-19 Vaccine Acceptance among Dental Professionals: A Multi-Country Survey | 2022 | Nassani MZ | 0 | 0 | 0 | 1 | 0 | 1 | 1 | 1 | 0 | 4 |
| Evaluation of COVID-19 Vaccine Refusal in Healthcare Workers | 2022 | Yahşi A | 0 | 0 | 1 | 1 | 0 | 0 | 1 | 1 | 0 | 4 |
| Evaluation of knowledge thought and attitudes of Health Services Vocational School students regarding COVID-19 vaccine applications | 2022 | Karadayı S | 0 | 0 | 1 | 0 | 0 | 1 | 1 | 1 | 0 | 4 |
| Factors Effecting COVID-19 Vaccine Refusal and Hesitation After Initiation of National Vaccination Program in Turkey | 2021 | Ünal O | 0 | 0 | 1 | 1 | 0 | 0 | 1 | 1 | 0 | 4 |
| Pandeminin Bir Yil Sonrasinda COVID-19 Asisina Toplumsal Yaklasim | 2022 | Durduran Y | 0 | 0 | 1 | 0 | 0 | 1 | 1 | 0 | 1 | 4 |
| Inactivated COVID-19 Vaccine Hesitancy Among Midwifery Students: a Prospective Online survey | 2022 | Turan A | 0 | 0 | 0 | 0 | 0 | 1 | 1 | 1 | 0 | 3 |
| Intentions to be Vaccinated Against COVID-19: The Role of Prosociality and Conspiracy Beliefs across 20 Countries | 2022 | Enea V | 0 | 0 | 0 | 0 | 0 | 1 | 1 | 1 | 0 | 3 |
|  |  |  |  |  |  |  |  |  |  |  |  |  |
| Q1: Was the sample frame appropriate to address the target population? | | | | | | | | | | | | |
| Q2: Were study participants sampled in an appropriate way? | | | | | | | | | | | | |
| Q3: Was the sample size adequate? | | | | | | | | | | | | |
| Q4: Were the study subjects and the setting described in detail? | | | | | | | | | | | | |
| Q5 Was the data analysis conducted with sufficient coverage of the identified sample? | | | | | | | | | | | | |
| Q6: Were valid methods used for the identification of the condition? | | | | | | | | | | | | |
| Q7: Was the condition measured in a standard, reliable way for all participants? | | | | | | | | | | | | |
| Q8: Was there appropriate statistical analysis? | | | | | | | | | | | | |
| Q9: Was the response rate adequate, and if not, was the low response rate managed appropriately? | | | | | | | | | | | | |
|  |  |  |  |  |  |  |  |  |  |  |  |  |
|  |  |  |  |  |  |  |  |  |  |  |  |  |
|  |  |  |  |  |  |  |  |  |  |  |  |  |
